# The Combination of Single-Cell and Next-Generation Sequencing Can Reveal Mosaicism for *BRCA2* Mutations and the Fine Molecular Details of Tumorigenesis

**DOI:** 10.3390/cancers13102354

**Published:** 2021-05-13

**Authors:** Alexandra Gráf, Márton Zsolt Enyedi, Lajos Pintér, Éva Kriston-Pál, Gábor Jaksa, Árpád Bálind, Éva Ezer, Péter Horváth, Farkas Sükösd, Ernő Kiss, Lajos Haracska

**Affiliations:** 1 HCEMM-BRC Mutagenesis and Carcinogenesis Research Group, Institute of Genetics, Biological Research Centre, 6726 Szeged, Hungary; graf.alexandra@brc.hu (A.G.); kiss.erno@brc.hu (E.K.); 2 Delta Bio 2000 Ltd., 6726 Szeged, Hungary; marton.enyedi@deltabio.eu (M.Z.E.); lajos.pinter@deltabio.hu (L.P.); kriston_pal.eva@deltabio.hu (É.K.-P.); jaksa.gabor@deltagene.hu (G.J.); 3 Lendület Laboratory of Microscopic Image Analysis and Machine Learning, Institute of Biochemistry, Biological Research Centre, 6726 Szeged, Hungary; balind.arpad@brc.hu (Á.B.); horvath.peter@brc.hu (P.H.); 4 Department of Clinical Oncology, Teaching Hospital Mór Kaposi, 7400 Kaposvár, Hungary; ezer.eva@kmmk.hu; 5 Institute for Molecular Medicine Finland (FIMM), University of Helsinki, 00014 Helsinki, Finland; 6 Department of Pathology, Faculty of Medicine, University of Szeged, 6720 Szeged, Hungary; sukosd.farkas@med.u-szeged.hu

**Keywords:** *BRCA2*, laser microcapture microscopy, tumor sequencing

## Abstract

**Simple Summary:**

Germline and somatic *BRCA1/2* mutations may define therapeutic targets and refine cancer treatment options. However, routine BRCA diagnostic approaches cannot reveal the exact time and origin of *BRCA1/2* mutation formation, and thus, the fine details of their contribution to tumor progression remain less clear. We established a diagnostic pipeline using high-resolution microscopy and laser microcapture microscopy to test for *BRCA1/2* mutations in tumors at the single-cell level, followed by deep next-generation sequencing of various tissues from the patient. To demonstrate the power of our approach, here we present a detailed analysis of an ovarian cancer patient, in which we describe constitutional somatic mosaicism of a *BRCA2* mutation. Characterization of the mosaic mutation at the single-cell level contributes to a better understanding of *BRCA* mutation formation and supports the concept that the combination of single-cell and next-generation sequencing methods is advantageous over traditional mutational analysis methods.

**Abstract:**

Germline mutations in the *BRCA1* and *BRCA2* genes are responsible for hereditary breast and ovarian cancer syndrome. Germline and somatic *BRCA1/2* mutations may define therapeutic targets and refine cancer treatment options. However, routine BRCA diagnostic approaches cannot reveal the exact time and origin of *BRCA1/2* mutation formation, and thus, the fine details of their contribution to tumor progression remain less clear. Here, we establish a diagnostic pipeline using high-resolution microscopy and laser microcapture microscopy to test for *BRCA1/2* mutations in the tumor at the single-cell level, followed by deep next-generation sequencing of various tissues from the patient. To demonstrate the power of our approach, here, we describe a detailed single-cell-level analysis of an ovarian cancer patient we found to exhibit constitutional somatic mosaicism of a pathogenic *BRCA2* mutation. Employing next-generation sequencing, *BRCA2* c.7795G>T, p.(Glu2599Ter) was detected in 78% of reads in DNA extracted from ovarian cancer tissue and 25% of reads in DNA derived from peripheral blood, which differs significantly from the expected 50% of a hereditary mutation. The *BRCA2* mutation was subsequently observed at 17–20% levels in the normal ovarian and buccal tissue of the patient. Together, our findings suggest that this mutation occurred early in embryonic development. Characterization of the mosaic mutation at the single-cell level contributes to a better understanding of *BRCA* mutation formation and supports the concept that the combination of single-cell and next-generation sequencing methods is advantageous over traditional mutational analysis methods. This study is the first to characterize constitutional mosaicism down to the single-cell level, and it demonstrates that *BRCA2* mosaicism occurring early during embryogenesis can drive tumorigenesis in ovarian cancer.

## 1. Introduction

Cancer is a genetic disorder caused by mutations of the susceptible genes, leading to the malignant transformation and clonal expansion of the tumor cells. The development of tumors is similar to the Darwinian evolution since modification of certain genetic properties such as inactivation of tumor suppressors appear gradually, providing selective advantages for the affected cells compared to the wild types [1,2,3]. Mutation or complete loss of the *BRCA1* and *BRCA2* genes, often referred to as driver mutations, are considered to be the first evolutionary steps during the development of a high percentage of breast and ovarian cancers, due to the key role of *BRCA* proteins within the maintenance of genome stability, achieved by multiple mechanisms [4,5,6,7] such as homologous recombination, a mechanism of DNA double-strand break repair [8,9,10,11].

We can distinguish between the inherited and somatic forms of *BRCA*-related mutations [12]. In the case of hereditary tumorigenesis, the *BRCA* mutant allele comes from one of the reproductive cells resulting in heterozygous somatic cells. The other, originally wild-type allele of the offspring is inactivated locally by somatic alterations during tumor development. In the somatic type of tumorigenesis, both *BRCA* alleles are inactivated through mutations appearing in the tumor cells [13]. The prevalence of germline *BRCA* mutations is 10–20% of all breast and ovarian cancer patients, while the proportion of somatic *BRCA* mutations is between 5 and 10% [14,15,16]. Since germline mutations can dramatically increase the risk of developing breast and/or ovarian cancer, the earliest identification of *BRCA* mutations is crucial for cancer prevention. The detection of both types of mutations from patients may provide essential information about the pathogenesis of their tumors [17]. There are targeted cancer therapies for both somatic and germline *BRCA*-mutant ovarian cancers; therefore, the evaluation of mutational patterns during diagnosis may help identify potential targets for specific drugs such as PARP inhibitors [9,18,19] and alkylating agents [20,21], and contribute to decision making, enabling more effective treatment strategies. However, the available DNA samples of different quantity and quality, the various types of mutations, and the emergence of new targeted cancer therapies all require the development of innovative diagnostic pipelines for *BRCA1/2* that integrate the multiple needs and offer more reliable methods for detecting hereditary and somatic mutations of the *BRCA* genes.

The identification of alterations in *BRCA1/2* genes represents a fundamental step in the early diagnosis and treatment of breast and/or ovarian tumors. However, the large size and the lack of mutational hotspots in these genes make traditional Sanger sequencing-based diagnosis, even for the diagnosis of germline *BRCA1/2* mutations, laborious and time consuming and, for somatic mutations, unreliable due to tumor heterogeneity. Next-generation sequencing (NGS) technologies have recently opened the possibility to analyze multiple DNA samples and to obtain large sequence datasets [17]. The availability of affordable benchtop NGS systems offered the possibility to transfer the *BRCA1/2* diagnostic workflow onto these high-throughput platforms, to improve and optimize the molecular diagnosis of mutational events in cancer.

A major limiting factor of the molecular diagnosis is the heterogeneity of cell populations isolated from tumor tissues [22,23,24,25]. Macrodissected tumor samples possess large degrees of phenotypic and genotypic cell-to-cell variability, which makes the evaluation of sequencing data challenging, since averaging the data of diverse populations of cells can lead to false conclusions if they mask the presence of underrepresented subpopulations. However, single-cell isolation by laser capture microdissection (LCM) can separate unique cell subpopulations with specific phenotypes without substantial disruption of the neighboring tissue while reducing contamination with other cell types. This type of sample isolation can provide contextual information and additional insights for data interpretation during genetic analysis [26].

To increase the accuracy and efficacy of microscopy-based image analysis, followed by single-cell isolation, we have recently developed a new pipeline for the genetic analysis of cell heterogeneity in various tissues [27]. Using partly this approach in this study, we show that the combination of LCM and NGS technologies further enhances the sensitivity of identification of rare mutations from trace amounts of tumor cells and can help reveal the fine molecular details of the emergence of *BRCA1/2* mutations and tumorigenesis.

## 2. Materials and Methods

### 2.1. Patient History and the Subjects of the Experiments

- Ovarian cancer patient

The patient is 66 years old, female. There is no other meaningful disease in her patient history. She has no siblings; her grandfather (father’s side) was diagnosed with cancer earlier (unspecified). The parents of the patient had no tumorous disease diagnosis. DNA was not available from any of the patient’s ascendants. The patient smokes, approximately half a box a day. The patient is moderately developed, 164 cm and 48 kg. In 2011, she visited her doctor with low-back pain and was diagnosed with ovarian serous adenocarcinoma WHO grade 2. (pT1c, peritoneal lavage: C5) in the Teaching Hospital Mór Kaposi, Department of Clinical Oncology, Kaposvár, Hungary. In the same year, she had a hysterectomy and bilateral adnexectomy. According to the protocol, TAX-BCP treatment was started after the surgery, but, due to side effects, the dosage was decreased. As the tumor showed regression, the treatment was ended. Later, based on clinical data, reinduction treatment was necessary. Since the end of the reinduction treatment, the patient is treated with Lynparza (Olaparib, 150 mg) only. First, she took 2 × 8 pills per day; later, the dosage was decreased to 2 × 6 pills per day. The patient’s condition has been stable since 2017. Celemics OncoRisk tumor panel (Celemics, P002) sequencing was performed on the patient’s tumor specimen; it showed no other affected gene from the 31 genes of the panel associated with tumorigenesis. Subsequently, *BRCA2* mutation determination and analysis were performed based on our study. As a preventive examination, the buccal mucosal tissue of the patient’s daughter was sequenced, and based on our results, the offspring did not inherit the *BRCA2* mutation. Informed consent was obtained from the patient.

- Analyzed tissue types from the cancer patient
  · FFPE block with ovarian serous adenocarcinoma WHO grade 2. for *BRCA 2* mutation analysis with a diagnostic purpose;
  · FFPE block with tumor-free ovarian tissue (chosen by an expert) from the cancer patient (called normal);
  · Blood (venous) sample from the cancer patient;
  · Buccal mucosal tissue (swab) from the cancer patient.
- Analyzed tissue type from the offspring of the patient
  · Buccal mucosal tissue.

All the laser capture microdissected cells were selected by a pathologist.

### 2.2. Overview of the Workflow

**Figure 1:**
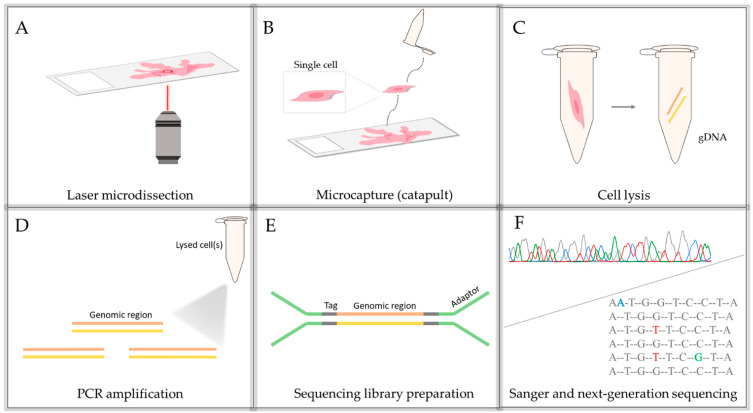
Schematic workflow for the single cells/small portion of cells: (**A**) selected cell(s) of interest are microdissected from the FFPE tissue based on the work of Brasko et al. [27]; (**B**) laser-dissected cells are catapulted into a PCR tube’s cap containing catapult buffer; (**C**) samples are lysed, the genomic DNA becomes accessible; (**D**,**E**) in a two-step PCR reaction, the genomic region of interest is amplified with universal tag sequences and Illumina specific adaptor sequences; (**F**) parallelly, Sanger and next-generation sequencing are performed on the amplicons.

In this study, we are analyzing tissue blocks and single cells/ cell clusters of 5–10 cells parallelly. The workflow for the small portion of cells is shown in Figure 1. For the cell cluster and single cell analysis, laser-capture microdissection was used. The chosen cells were catapulted into the caps of 200 µL microcentrifuge tubes. The cells were chosen by an expert. After the microcapture, the cells were lysed and the analyzed *BRCA2* genomic region was amplified together with the universal tags and the Illumina-specific adaptors. The samples were analyzed by Sanger and next-generation sequencing as well.

### 2.3. gDNA Isolation from Tissue and Single Cells/Bulks of 5–10 Cells

For isolation of genomic DNA from macrodissected tissue blocks and whole blood samples, the phenol-chloroform protocol was used [28]. Laser-capture microdissected (LCM) cells were captured in 5 µL catapult buffer (10 mM EDTA, 2 mM Tris pH8, 0.5% Igepal (Cat.no.: I8896, Sigma Aldrich, St. Louis, MO, USA). The blood samples for LCM were fixed with 96% ethanol. Mature, peripheral lymphocyte cells were chosen by an expert; the lymphocyte cells were not subtypified. After LCM, we added 0.5 µL Proteinase K (Cat.no.: 19131, Qiagen, Hilden, Germany) (1 mg/mL) to the samples and incubated at 60 °C for 20 min, followed by 3 min at 98 °C. Next, a two-step amplification reaction was carried out in 20 µL volume.

### 2.4. Amplification and Sequencing of the Examined BRCA2 Region

The genomic region containing the *BRCA2* c.7795 position, which we previously found to be mutated by *BRCA1/2* all-exon sequencing, was amplified in a two-step PCR. In the first PCR, we used a 10 µM *BRCA2*-specific primer paired with universal tag sequences, resulting in a 148-bp product. PCR conditions were as follows: 1X PCR buffer, 2.0 mM MgCl_2_, 2.5 mM dNTPs, and 1 unit of AmpliTaq Gold DNA Polymerase (Thermo Scientific, cat.no.: 4311806, Waltham, MA, USA). Thermal cycler conditions were as follows: 95 °C for 2 min, 25 cycles of 95 °C for 15 s, 61 °C for 30 s, 72 °C for 20 s, and finally, 1 min at 72 °C. The sequences of the oligos, used in the first PCR are the following: GGATAGTCAAGGTCAGGTGGGCTCATACCCTCCAATGATGGAAAG (Fw), GACGCTGGAATGTAACAATGGGAGAAGAAAGAGGGATGAGGGAATAC (Rev). In the second PCR, 1 µL from the first amplification reaction was used as a template with primers complementary to the universal tag sequence carrying Illumina-specific adaptor sequences at the 5′ ends. Thermal cycler conditions were as follows: 95 °C for 2 min, 30 cycles of 95 °C for 10 s, 70 °C for 30 s, 72 °C for 20 s, and finally, 1 min at 72 °C. The second PCR resulted in a 288-bp product. Reactions were run on a 2% agarose gel for amplification quality control. Successfully amplified samples were sequenced on a Sanger or an NGS platform. NGS samples were quantified using the Qubit HS quantification method (Thermo Scientific). Illumina sequencing was carried out on the Illumina MiniSeq system with MiniSeq Mid Output Kit 300-cycles (FC-420-1004), following the manufacturer’s instructions.

### 2.5. Data Analysis

Sequencing data were analyzed using the CLC Genomics 3.6.5 bioinformatics software (Qiagen).

Reads were mapped to BRCA2-206: RefSeq. NM_000059, Transcript ID: ENST00000544455.5, and variant calling was performed to identify mutant and wild-type allele percentages in the c.7795 position of BRCA2. The average read number for the ethanol fixed lymphocyte cells was 18.379 (min.: 3994, max.: 35.230), and for the FFPE tissue samples, the average was 287 (min.: 70, max.: 636). The read numbers are consistent with the coverage as we were counting only with those reads that covered the mutation carrying region.

## 3. Results

### 3.1. Bulk Tissue Sample Sequencing—Comparison of Sanger Sequencing and NGS

First, we examined formalin-fixed, paraffin-embedded (FFPE) tissue samples from the ovarian tumor of the patient by *BRCA1/2* all-exon sequencing and found a point mutation (G>T) in the c.7795 position in the exon 16 of the *BRCA2* gene. This G>T substitution generates a stop-codon, which results in the premature termination of translation and as a consequence in a truncated *BRCA2* protein. The c.7795G>T, p.(Glu2599Ter) mutation was previously identified in other hereditary breast and ovarian cancer patients, and it is considered to be pathogenic (*BRCA* Exchange Database) [29]. Next, to gain a deeper insight into this *BRCA2* point mutation, macrodissected blocks of tumorous and nontumorous tissues were used. To compare the reliability of mutation detection, Sanger sequencing and NGS were executed parallelly. NGS results showed a 77–78% appearance of the mutant T nucleotide instead of the wild-type G at position 7795 in the tumor samples. By comparing the size of corresponding peaks, we could estimate approximately the same ratio in the case of Sanger sequencing (Table 1). In a later phase of our experiments, buccal mucosal swab samples were examined the same way. Interestingly, we found the same G/T transition in approximately 20% of the NGS reads, which differs from the 50% expected for heterozygous alleles. This distorted ratio of genotypes may indicate that the patient is not a heterozygote for the *BRCA2* c.7795G>T, p.(Glu2599Ter) mutation but rather has a mosaic genotype. To prove our theory, the tumor-free environment of the ovary as “normal tissue” was examined, where the same, closely 20% of the mutated T alleles could be detected by both sequencing methods. Finally, we processed a blood sample from the patient, where again the same approximate ratio of G/T transition was found. Based on these deep sequencing results of the three different tumor-free tissue samples, we could conclude that the patient shows mosaicism instead of heterozygosity.

In addition, we were able to analyze the buccal mucosal swab sample of the patient’s daughter, where we could not detect the mutant T nucleotide, suggesting that this mutation was not inherited by the offspring of the patient.

### 3.2. Decreasing the Cell Number and Taking a Deeper Insight—Sanger and NGS Comparison on Microdissected Single Cells/Clusters of 5–10 Cells

By conventional sequencing of macrodissected tissue blocks, we received an overview of the average genetic background of the examined tumor sample; however, this approach alone was not able to answer questions about the cell subpopulations within the tumor, and it could not reveal rare variants.

To circumvent these limitations, microdissected single cells or cell clusters (5–10 cells) were used as starting material. By Sanger sequencing of nontumorous ovary tissue samples, we could identify both heterozygous mutant and wild-type single cells (Table 2, Table S1). From cell cluster samples (5–10 microdissected cells), we estimated that approximately 30% of the cells carried the mutant T nucleotide. Contrary to the nontumorous samples, in the cell cluster sample, we found exclusively the mutant T instead of G, suggesting previous loss of heterozygosity (LOH) events within the tumor.

By next-generation sequencing, twelve single cells from the nontumorous tissue of the patient’s ovary and five cell clusters of 5–10 cells from the tumor tissue (and one cluster of normal tissue) were examined (Table 3). Single tumor cells were not examined since results from these cells would not provide additional information to our work since our major goal was to validate our pipeline through a concrete case of *BRCA2* somatic mosaicism. In the sample that contained 5–10 nontumorous ovarian cells (Norm_cl-01), a near heterozygous genotype can be seen, while 42% of the normal single cells (5/12) exhibited wild-type and 16% heterozygous (2/12) genotypes. Surprisingly, in several cases (5/12), we obtained reads with distorted G/T ratios from which the genotyping of the cells was uncertain. For example, in the Norm-04 sample, the mutant T allele was found in 79% of the reads, while 21% of the reads carried the wild-type G nucleotide. These distorted ratios raise the question of where these abnormalities come from. One source of error could be the laser microdissection when the sample contains more than one cell. The other possibility could be defective PCR amplification; PCR reactions from single cells are often compromised by the phenomenon called allele drop out, where one of the alleles is more preferred as a template by the polymerase during the first few amplification cycles. As a result of such amplification, one of the alleles will be the source of the majority of the reads. Consequently, heterozygous cells can be evaluated as homozygous [30]. In the case of bulk samples, it is not only the amplification that can be the source of errors. It is also possible that cells with one of the genotypes are chosen mostly during the microdissection, which shifts the ratio of sequencing reads into one direction artificially, as in the case of the Tum_cl-02 and Tum_cl-05 tumor samples, where the low ratio of the mutation is unexpected.

### 3.3. Amplificability and Sequencability of FFPE Samples

Formalin-fixed, paraffin-embedded (FFPE) tissues are routinely used in molecular oncology diagnostics. However, extraction of genomic DNA of sufficient quality and quantity from FFPE samples is always challenging, since formalin fixation generates crosslinks between proteins and nucleic acids and DNA strand breaks, which negatively affect the amplification of the chosen genomic region of the cells. Laser microdissection and the process of cell catapulting can further decrease the success rate of amplification and sequencing as compared to non-FFPE or nonlaser-dissected samples. Since the amount of the starting material is reduced by using single cells or clusters of 5–10 cells, losing one cell during the process has a higher impact on the result, compared to the larger starting material. In our experiments, single cells and clusters of cells from normal (nontumorous ovarian) tissue and clusters of tumor cells were used (Table 4). From both the normal tissue and tumor cell clusters, 50% of the samples, while in the case of single normal cells, 12 out of 20 (60%) were suitable for amplification. We could sequence all of these PCR fragments, which indicates that successful PCR amplification is the rate-limiting step in our workflow.

### 3.4. Examination of Single Lymphocyte Cells

To determine the *BRCA2* genotype of the cancer patient from an independent somatic tissue and to compare the efficacy of our single-cell isolation and analysis pipeline from a non-FFPE tissue, 40 peripheral, mature, unsubtypified lymphocyte cells derived from 96% ethanol-fixed blood smears were analyzed. In the case of lymphocyte cells, only single cells were analyzed due to the more efficient PCR amplification, compared to FFPE samples. From these cells, 16 (40%) were suitable for PCR and 13 for NGS (Table 5).

For the amplification, we tested two types of polymerase enzymes: 11 were amplified with Taq polymerase, while 5 with Phusion polymerase. Sequencing reactions of DNA fragments amplified with the Taq polymerase were successful in 73% of the cases, while with Phusion polymerase the success was 100%. According to the NGS results, 6 of the 13 sequenced cells were wild type (62%), while three showed an almost clear heterozygous genotype (23%). Two samples (Lymp_Taq-01 and Lymp_Phu-02) showed distorted G/T ratios, which were unexpected for single diploid cells (Table 6). These 88%-12% and 4%-96% ratios could be generated by the phenomenon of allele drop out, as mentioned above, or by the errors of the polymerases. Interestingly, in two lymphocytes (15%), only the mutant T nucleotide was found, indicating homozygous *BRCA2* genotype, which is unexpected for nontumorous cells.

## 4. Discussion

Cancer is one of the leading causes of death worldwide. As a genetic disorder, changes within the DNA are responsible for the transformation and clonal expansion of the cells. These changes can offer selective advantages for the affected cells, such as the inactivation of the well-known tumor suppressor genes *BRCA1* and *BRCA2*, which play a key role in the development of breast and ovarian cancers. These genes have an important role in the maintenance of genome integrity through the DNA double-strand break repair pathway, the homologous recombination repair. As a result of the inactivation of these tumor suppressor genes, homologous recombination repair cannot function, and this results in mutation accumulation and also in the cells taking a step toward tumor progression.

In *BRCA* mutation-related tumorigenesis, inherited and sporadic-type cancers can be distinguished. According to a general view, in the case of inherited BRCA tumor progression, one of the two alleles of wild-type cells carries a germline mutation, while the other allele is inactivated within the tumor during the course of the tumor evolution. In the case of sporadic BRCA tumorigenesis, both alleles are inactivated in a somatic way during tumor evolution. The prevalence of mosaic BRCA mutations in hereditary breast and ovarian cancer seems to be low. Until now, only a few cases of de novo *BRCA1/2* mutations have been described outside tumors, and most of them show heterozygous genotypes [31,32,33]. To the best of our knowledge, only one patient with BRCA2 constitutional mosaicism has been described in the literature [34]; however, although mosaicism for BRCA1/2 mutations seems to be rare, this and the mentioned study demonstrate that low-level mosaic mutations can contribute to the etiology of breast and ovarian cancer susceptibility. Since nowadays detailed genetic determination experiments involving noncancer tissue types are not used in routine cancer diagnostics, further such studies are required to reveal the frequency of de novo BRCA1/2 mutations and their contribution to carcinogenesis.

Nowadays, effective personalized cancer therapies are available; therefore, timely identification of mutations and their origin is of great importance. Here, we show a comparative method for genetic analysis of tumor and nontumorous samples. We believe that if single-cell isolation by laser capture microdissection were as routine as next-generation sequencing, it could help decision making in everyday cancer diagnostics since it could provide more detailed information about the genetic background of the different tumor cell subpopulations, enabling the mapping of the tumor heterogeneity of the examined sample. For example, determination of the presence and ratio of chemotherapy-sensitive and -resistant cells in advance may lead to more successful targeted chemotherapeutic regimens. With the examination of various tissues, laser-dissected cell clusters consisting of 5–10 cells, and single cells with Sanger as well as NGS, we are able to gain a deeper insight into the composition of tumor-free tissues and also the tested tumor and to generate more precise information about the genetic origin of the tumor.

We provide a deeper insight into our method through the analysis of a cancer patient with bilateral ovarian serous adenocarcinoma WHO grade 2. pT1c3 (FIGO IC3). In our experiments, ovarian tumor tissue, tumor-free environmental (normal) tissue, buccal mucosal tissue, and blood were analyzed. Parallelly, macrodissected tissue blocks and laser microdissected single cells/ small cell clusters were used. This way of processing enabled us to compare the results of the macro- and microdissected samples with two different types of analysis: Sanger and next-generation sequencing. As our results show, we detected 77–78% mutated T alleles instead of G in the nucleotide position 7795 in FFPE tumor tissue blocks (Table 1), suggesting that the majority of the cells within the tumor lost their wild-type *BRCA2* alleles. The sequence data of the normal FFPE tissue block, the blood, and the buccal mucosal tissue show that about one-fourth of the examined nontumorous cells are heterozygous. These results suggest that this patient did not carry an inherited heterozygous *BRCA2* mutation in the nucleotide position 7795 (that would result in an approximate 50–50% of mutant and wild-type alleles) but rather showed a mosaic pattern. To the best of our knowledge, this is the first study in which *BRCA2* mosaicism was examined via the combination of next-generation sequencing and single-cell analysis.

**Figure 2:**
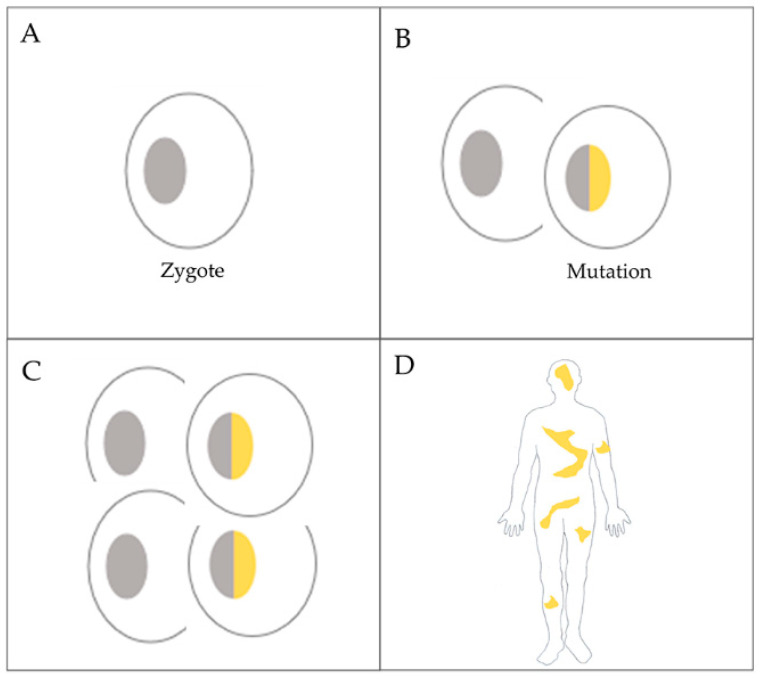
Schematic representation of the theoretical formation of BRCA2 mosaicism based on our results: (**A**) fertilized egg cell; (**B**) in the two-cell stage (or early in embryonic development), a G to T mutation in position 7795 of *BRCA2* occurs, generating a heterozygous cell; (**C**) stemming from this single-mutated cell and wild-type cell(s), cell divisions result in a situation when every second cell will be heterozygous; (**D**) in the developed human body, cells will carry the mutated T allele with an approximate 25% frequency.

Since samples were not available from the patient’s parents, we cannot absolutely exclude the phenomenon of revertant somatic mosaicism, where spontaneous correction of a pathogenic mutation occurs. However, in the case of *BRCA1/2*, this does not seem to be a common phenomenon [35], contrary to mutations in highly proliferative tissues such as in heritable skin diseases [36,37]. Based on our results, together with a slight chance of revertant mosaicism, the final 20–25% frequency of the mutated T nucleotide suggests that the mutation may have occurred very early in development, theoretically maybe in the two-cell phase of the developing embryo, assuming that the division dynamics of the wild-type and heterozygous cells are the same (Figure 2). We were able to examine the buccal mucosal swab of the patient’s daughter, and it showed 100% G in the nucleotide position 7795 of *BRCA2*. As she is the only offspring of the patient, we have no information about the heredity pattern of this mutation.

As a final summary, we collected all the data from the micro- and macrodissected samples we were able to examine. As demonstrated in Table 7, a 10–20% difference appeared between the G/T ratios of macro- and microdissected sample results generated by NGS. This shows the importance of decreasing the starting material and using single cells or cell clusters since it can provide a more precise and more informative view about the genetic characteristics of both the tumor and the cancer-free tissue of the patient.

By comparing different sample-isolation and sequencing methods, we were able to determine the genetic status of the *BRCA2* c.7795G>T mutation of the patient, and we could prove that this mutation shows a mosaic pattern rather than heterozygosity. By combining single-cell microdissection and NGS, we were able to overcome the limitations of Sanger sequencing and the use of bulks of heterogenic cells. Although our approach is more expensive, this can be a promising methodological solution for similar studies [38]. From the analysis of the NGS data of 16 single lymphocyte cells, we could clearly detect in some cases the appearance of the mutated T allele without the presence of the wild-type allele (G). This suggests that possibly homozygous *BRCA2* mutant cells can appear not just within the tumor, but outside of it as well, in nontumorous environments such as the vascular system. Although the allele-specific amplification could cover the heterozygous state of the cell, this is improbable due to the 18.000× average coverage in the case of the single lymphocyte cells. The presence of these mutant cells is unexpected since the *BRCA2* homozygous mutation was known to be lethal, except for the case of loss of heterozygosity within the tumor. However, based on these results, our suggestion is that the homozygous *BRCA2* mutation is lethal only at the organism level, which leads to embryonic lethality but not at the single-cell level, as demonstrated here.

## 5. Conclusions

In this study, we developed a method to reveal the fine molecular details of tumorigenesis by combining single-cell analysis and next-generation sequencing. To show the power of our approach, we used it to compare the *BRCA2* mutational status of tumor samples with several nontumorous tissues from an ovary cancer patient. We proved that the patient showed a mosaic pattern in the case of the *BRCA2* c.7795G>T mutation and, based on our results, we conclude that this mutation occurred de novo, during early embryonic development. Previous studies show that in the zygote, alleles with inherited inactivating *BRCA2* mutations can only be present in the heterozygous stage since, in the homozygous stage, they cause lethality. However, our single-cell sequencing data revealed that homozygous *BRCA2* mutant lymphocytes can exist in the peripheral blood of the patient, suggesting that at the single-cell level, this homozygous mutation does not have that high of an impact on the functioning of the organism.

## Figures and Tables

**Table 1:** Summary of the results of the Sanger and next-generation sequencing on different tissues from the patient (tumor, buccal mucosa, blood, and tumor-free “normal” ovary tissue surrounding the tumor) and her daughter (buccal mucosa).

Tissue Type	Sanger Sequencing (Yellow Box Indicates Position 7795 of *BRCA2*)	Next-Generation Sequencing (% of Mutant T Instead of Wild-Type G at Position 7795 of *BRCA2)*
Tumor 1	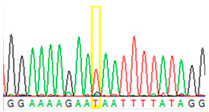	78%
Tumor 2	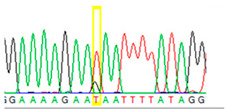	77%
Buccal musoca 1	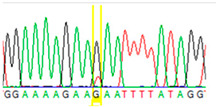	23%
Buccal musoca 2	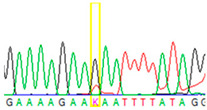	21%
Normal	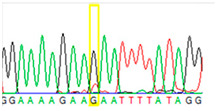	17%
Blood	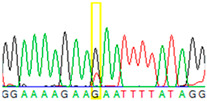	26%
Buccal mucosa (daughter)	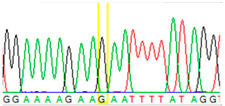	0%

**Table 2:** Examples from the Sanger sequencing results of the microdissected single cells and cell cluster samples.

Sample Type (Approximate Number of Cells)	Sanger Sequencing (Yellow Box Indicates Position 7795 of *BRCA2*)	Presenting Allele(s) (At Position 7795 of *BRCA2*)
Normal (5–10 cells)	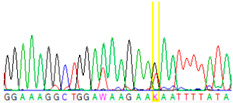	G/T
Normal (single cell)	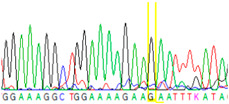	G
Normal (single cell)	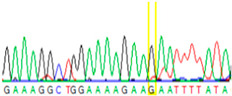	G
Normal (single cell)	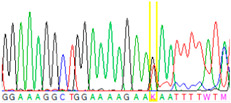	G/T
Normal (single cell)	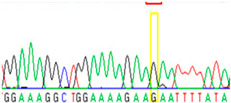	G
Normal (single cell)	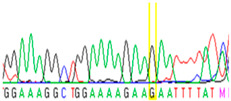	G
Tumor (5–10 cells)	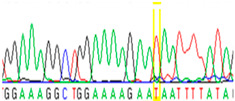	T

**Table 3:** NGS results of normal and tumor samples with single cells and clusters of 5–10 cells.

ID	Tissue/Cell	T	G
Norm-01	Normal 1	-	100%
Norm-02	Normal 1	7%	92%
Norm-03	Normal 1	9%	90%
Norm-04	Normal 1	79%	21%
Norm-05	Normal 1	57%	42%
Norm-06	Normal 1	94%	5%
Norm-07	Normal 1	-	100%
Norm-08	Normal 1	-	100%
Norm-09	Normal 1	53%	46%
Norm-10	Normal 1	-	100%
Norm-11	Normal 1	-	100%
Norm-12	Normal 1	7%	92%
Norm_cl-01	Normal 5–10	43%	57%
Tum_cl-01	Tumor 5–10	82%	18%
Tum_cl-02	Tumor 5–10	2%	97%
Tum_cl-03	Tumor 5–10	87%	12%
Tum_cl-04	Tumor 5–10	99%	-
Tum_cl-05	Tumor 5–10	4%	95%

**Table 4:** Summary of the success rate of amplification and sequencing of the analyzed *BRCA2* genomic region from FFPE samples.

Tissue Type	Cell Number	Sample Number	Amplification		Sequencing	
FFPE	pcs	pcs	pcs	%	pcs	%
Normal tissue	5–10	2	1	50	1	100
Normal tissue	1	20	12	60	12	100
Tumor tissue	5–10	10	5	50	5	100

**Table 5:** Amplificability and sequencability of single lymphocyte cells of the cancer patient.

Tissue Type	Cell Number	Sample Number	Amplification		Sequencing	
	pcs	pcs	pcs	%	pcs	%
Lymphocyte	1	40	16	40	13	80

**Table 6:** Summary of the genotypes of the 13 sequenced single lymphocyte cells.

Lymphocyte ID	G	T
Lymp_Taq-01	88%	12%
Lymp_Taq-02	99%	-
Lymp_Taq-03	99%	-
Lymp_Taq-04	99%	-
Lymp_Taq-05 *	-	-
Lymp_Taq-06 *	-	-
Lymp_Taq-07	65%	34%
Lymp_Taq-08	-	99%
Lymp_Taq-09	99%	-
Lymp_Taq-10 *	-	-
Lymp_Taq-11	99%	-
Lymp_Phu-01	99%	-
Lymp_Phu-02	4%	96%
Lymp_Phu-03	58%	41%
Lymp_Phu-04	-	99%
Lymp_Phu-05	34%	65%
Sum	64.80%	34.30%

* Unsuccessful NGS

**Table 7:** Final summary of the sequenced single cells/bulks of 5–10 cells, compared to the macrodissected samples from the same tissue with G/T ratios.

Tissue Type		Microdissection			Macrodissection
		Cell Number/ Reaction	Number of Samples	Genotype	
				Mutant Allele	
				T (G)	
FFPE	Ovarian normal tissue	1	12	26%	17%
	Ovarian tumor tissue	5–10	5	55%	78%
Native	Lymphocyte	1	13	35%	26%

## Data Availability

The data presented in this study are available on request from the corresponding author. The data are not publicly available due to privacy restrictions.

## Supplementary Materials

The following are available online at https://www.mdpi.com/article/10.3390/cancers13102354/s1, Table S1: The result of Sanger sequencing in the rest of the single normal cells and bulks of 5–10 tumor cells.
