# Supplementary material for: The Combination of Single-Cell and Next-Generation Sequencing Can Reveal Mosaicism for BRCA2 Mutations and the Fine Molecular Details of Tumorigenesis
Source: Cancers (Basel). 2021 May 13;13(10):2354. doi: 10.3390/cancers13102354 (PMC8153129; doi:10.3390/cancers13102354)
Supplement: Supplementary file 1 [file cancers-13-02354-s001.zip › cancers-1182682-supplementary.pdf]

cancers
MDPI
Supplementary Materials

# The Combination of Single-Cell and Next-Generation Sequencing Can Reveal Mosaicism for *BRCA2* Mutations and the Fine Molecular Details of Tumorigenesis

**Alexandra Gráf, Márton Zsolt Enyedi, Lajos Pintér, Éva Kriston-Pál, Gábor Jaksa, Árpád Bálind, Éva Ezer, Péter Horváth, Farkas Sükösd, Ernő Kiss, and Lajos Haracska**

Table S1. The result of Sanger sequencing in the rest of the single normal cells and bulks of 5-10 tumor cells

| Sample type (Approximate number of cells) | Sanger sequencing (yellow box indicates position 7795 of BRCA2) | Presenting allel(s) (At position 7795 of BRCA2) |
|---|---|---|
| Normal (single cell) | 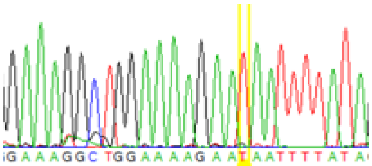 | T |
| Normal (single cell) | 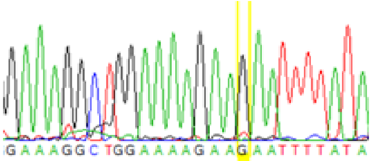 | G |
| Normal (single cell) | 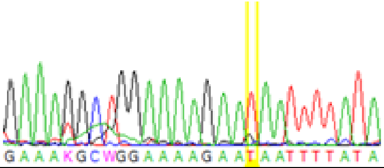 | T |
| Normal (single cell) | 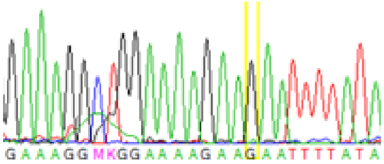 | G |
| Normal (single cell) | 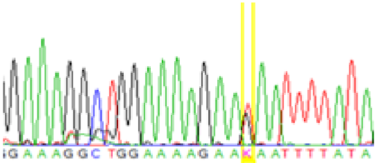 | G/T |
| Normal (single cell) | 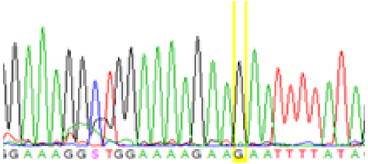 | G |

| | | |
|---|---|---|
| Normal (single cell) | 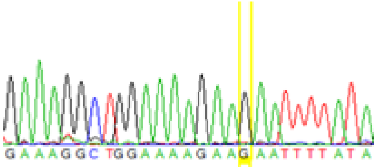 | G |
| Tumor (5-10 cells) | 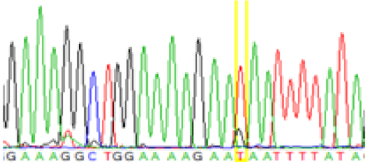 | G/T |
| Tumor (5-10 cells) | 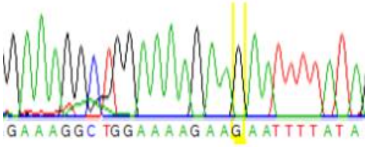 | G |
| Tumor (5-10 cells) | 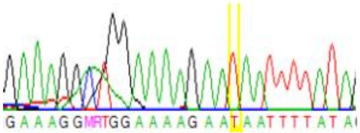 | T |
| Tumor (5-10 cells) | 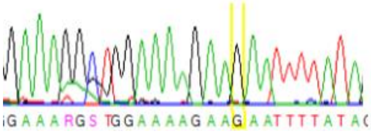 | G |
